# Social contributions as risk factors for readmissions after lung transplantation: Clinical and financial implications

**DOI:** 10.1016/j.jhlto.2025.100300

**Published:** 2025-05-26

**Authors:** Catherine Lu Dugan, Margaret V. Kudlinski, Sivagini Ganesh, Graeme Rosenberg, Takashi Harano, Sean Wightman, Scott Atay, Anthony W. Kim, Brooks V. Udelsman

**Affiliations:** a Keck School of Medicine of USC, Los Angeles, CA; b Surgery, Keck Medicine of USC, Los Angeles, CA; c Medicine, Keck Medicine of USC, Los Angeles, CA

**Keywords:** Healthcare disparities, Social determinants of health, Lung transplantation, Psychosocial

## Abstract

**Introduction:**

Lung transplant is associated with a 60%–80% 1-year post-transplant readmission rate. Social contributors represent potentially modifiable risk factors for readmission. We compared the clinical and financial of implications of readmissions associated with and without social factors.

**Methods:**

Retrospective single-center study of lung transplant patients surviving to discharge between 2/2/2013 and 4/11/2023. Two reviewers categorized 1-year readmissions into two groups: social (eg, housing instability or rejection due to medication non-compliance) and non-social (eg, pancreatitis). Sociodemographics, transplant indications, Stanford Integrated Psychosocial Assessment for Transplant scores, lung allocation score, pre-operative hospitalization status, in-hospital post-operative course, and readmission costs were compared between patients with and without a social readmission.

**Results:**

Among 192 transplants (109 double, 83 single), there were 436 1-year readmissions, including 33 social readmissions. Reviewer inter-rater reliability was >95% and Kappa was 0.91. A social readmission occurred in 21 (11%) patients, and 9 of these patients had multiple social readmissions. A social readmission was either the first or second readmission for 81% of these patients. Patients with a social readmission had a greater median number of readmissions (4 vs 2; *p* < 0.001) and were associated with longer length of stay (8 vs 5 days; *p* < 0.004), increased hospital costs ($23,813 vs $14,245; *p* = 0.04), and decreased margin (-$6145 vs $2287; *p*<0.001).

**Conclusions:**

Social readmissions represent a burden on patients and health systems. There is a strong association between social readmissions and increased costs, length of stay, and number of readmissions. Outpatient investment in patients with first-time social readmissions may improve outcomes and decrease healthcare costs.

## Background

Lung transplant is a complex operative procedure associated with high morbidity. Between 60% and 80% of post-transplant patients are readmitted within 1 year.^1^ Multiple causes have been cited for readmission, including rejection, infection, and cardiovascular deterioration.^2^ Altogether, these readmissions place an enormous burden on the healthcare system and are a contributor to poor quality of life among patients.

In addition to medical complications leading to readmission, social factors may play a role in postoperative hospitalization.^3^ Coping with life after lung transplantation can be difficult for patients who are often debilitated preoperatively and for their caregivers, as it requires long periods of recovery.^4^ Social contributors, such as inability to afford medications, represent potentially modifiable risk factors for readmission.

Quantifying the degree to which social factors contribute to each unique readmission after lung transplant is important. Reducing unexpected readmissions is a mandate of the Affordable Care Act and therefore tied to compensation, and it is associated with high-quality care.^5^ Determining the contribution of social factors may help to allocate resources and prevent social readmissions from occurring. Our aims were to assess the prevalence of social readmissions after lung transplant and to assess the frequency, cost, and length of stay associated with these readmissions.

Based on weekly multidisciplinary meetings in which all inpatients are discussed, we hypothesized that social factors would be partially responsible for greater than 25% of 1-year readmissions for patients status post lung transplant and that these readmissions would be associated with significantly greater in-hospital costs.

## Materials and methods

### Patients and data set

We retrospectively reviewed patients >18 who received a first-time lung transplant between 2/2/2013 and 4/11/2023 and survived to discharge within a single-center institutional database. Patients who received a multi-organ transplant were excluded from analysis. Pre-transplant patient sociodemographic information, body mass index, and comorbidities were collected. In addition, limitations to activities of daily living and living status (alone or with family/caregivers) were recorded. Prior to transplant, the Stanford Integrated Psychosocial Assessment for Transplant (SIPAT) was performed, and the score was collected. The SIPAT, first developed in 2012, assess the social factors that predict patients’ post-transplant outcomes by evaluating patient risk factors in four domains: patients’ readiness, social support, psychological stability, and substance abuse.^6^ SIPAT scores range from 0 to 69, where those patients’ scoring 0-6 are considered excellent candidates; 7-20 good candidates; 21-39 minimally acceptable candidates, and those scoring 40+ are high risk and poor candidates.

Transplant and operative variables included indication for transplant, type of transplant (ie, double or single), lung allocation score (LAS), need for transfusion, and use of extracorporeal membrane oxygenation. Postoperative variables included index hospitalization length of stay, whether reoperation was required, whether the patient suffered delayed graft failure or reintubation, new onset diabetes, pneumonia, acute respiratory distress syndrome, renal insufficiency, urinary tract infection, thromboembolic events, ileus, gastroparesis, new atrial or ventricular arrhythmia, cardiogenic shock, or stroke. Perioperative variables included whether the patient was hospitalized prior to transplant and discharge disposition.

### Exposure: social readmission

All readmissions within 1-year of transplant were recorded. Readmission was defined as an unplanned hospitalization lasting greater than 24 hours. The study site does not have a self-contained emergency department; however, anyone transferred who stayed >24 hours was marked as a readmission. Patients who present to outside hospital emergency departments with a history of transplant at our institution are preferentially transferred to our hospital. All patients admitted via transfer or direct hospital admission were captured.

Every readmission was evaluated for evidence of a social component based on preset definitions (Table 1). These definitions were based on previous work by Andrew and Powell (2016) who defined a social admission as an event where acute medical problems were not contributing, but rather social factors were the cause of admission.^7^ We used a modified version of this definition to define a social admission as an admission in which the underlying root cause is related to social determinants rather than medical. For example, if a patient presents for rejection because they stopped taking their medications due to an inability to afford them, this would be classified as a social readmission. In contrast, a patient who developed rejection but was compliant with medications and medical instruction would be classified as a non-social readmission. Social drivers included insurance loss (eg, inability to afford medication), lack of support personnel (eg, loss of caregiver), care non-compliance (eg, refusal of home health or physical therapy), mediation non-compliance (eg, refusal to take immunosuppressants), and maladjustment (relapse of substance use disorders, overwhelming depression/anxiety related to transplant). Pre-study validation of the definition for social readmission was completed by two independent reviewers, and the definition was refined until a 95% concordance between reviewers was achieved. These definitions were discussed with transplant team members, including surgeons, transplant pulmonologists, and social workers to insure concordance and validity.

**Table 1:** Readmission Classification Definitions

Category	Definition	Example
Non-social	Patient developed a medical complication unrelated to access, care, or treatment at home. While social issues may be coincident, they did not directly contribute to the readmission.	Stroke, myocardial infarction, bowel obstruction, pneumonia.
Social	Patient developed a medical complication that was partially or fully related to a social issue.	Developed rejection secondary to immunosuppression mismanagement, caregiver burnout, inability to perform ADLs independently and lacking support.

ADLS, activities of daily livings.

Classification was determined based on chart review, including clinician notes and social work evaluations conducted by the lung transplant team social worker during each in-patient encounter. Review of each readmission was performed by two research staff. Any disagreement between reviewers was reviewed by an attending surgeon on the study staff. This involved an intensive process of chart review, and all classifications of readmissions were assessed for inter-rater reliability through the Kappa (k) coefficient.

### Outcomes: number of readmissions, length of readmission, and cost of readmission

Total number of readmissions within 1-year of transplant was recorded. Length of stay (in days) and cost were analyzed for each readmission. Cost data (net revenue, variable cost, and contribution margin) were obtained for each encounter directly from the hospital billing department. Net revenue was defined as the amount of money the hospital received from the payor (ie, insurance company, government, self-pay). Variable cost was defined as the cost of nursing care, room and board, and supplies but did not include physician or advanced practice provider salaries. Contribution margin was defined as the amount of money the hospital gained or lost from a given readmission. It was calculated as the net revenue minus the total cost. Cost data is reported in a manner consistent with equator network guidelines.^8^

### Statistics

Inter-rater reliability for categorizing a readmission as social or non-social was measured by Kappa (*k*) coefficient. Statistical analysis was conducted at the patient level and the readmission level. At the patient level, two groups were compared: the social group, comprised of patients with at least one social readmission, and the non-social group, comprised of patients with no social readmissions. At the readmission level, social readmissions were compared with non-social readmissions.

At the patient level, bivariate analysis of patient sociodemographic information, transplant characteristics, post-operative hospitalization, number of readmissions, length of stay per readmission, and hospital costs of each readmission comparing the social group versus the non-social group was conducted. At the readmission level, length of stay and hospital cost were compared between social and non-social readmissions and expressed as mean with an interquartile range. Statistically significant differences in these distributions were identified using the *Χ*^2^ test or Fisher exact test for categorical variables and the *t*-test or Mann-Whitney-*U* test for continuous variables. To explore differences in the timing of social and non-social readmissions within 1 year, a time-to-event analysis was performed with death as a censoring event. Other potential censoring events, such as retransplant and loss to follow up did not occur in the study’s 1-year time-period.

For all analyses, two-sided *p*-values < 0.05 were considered statistically significant. Computations were carried out using Stata software, version 15.1 (StataCorp, College Station, Texas).

## Results

### Patient characteristics

**Figure 1:**
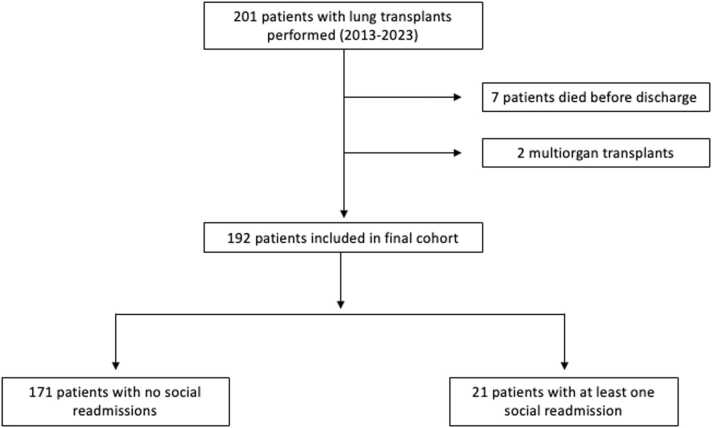
STROBE diagram.

A total of 201 patients underwent lung transplantation between 2/2/2013 and 4/11/2023. Seven patients died prior to discharge, and two multi-organ transplant recipients were excluded from analysis (Figure 1). Of the 192 included transplant patients, 100 (52.1%) were female, and the average age was 53.5 years. The most common indication for transplant was interstitial lung disease (*N* = 111, 57.8%) followed by COPD (*N* = 40, 20.8%) (Table 2). The average postoperative length of stay was 16 days, after which 139 (72.4%) were discharged home (vs rehab) (Table 3).

**Table 2:** Patient Characteristics

Factor	Total *N* = 192	No Social Readmission *N* = 171	Social Readmission *N* = 21	*p*-value
Age, mean (SD)	53.46 (13.60)	53.48 (13.11)	53.29(17.48)	0.95
Female	100 (52.1%)	91 (53.2%)	9 (42.9%)	0.37
BMI, mean (SD)	25.27 (4.42)	25.38(4.50)	24.38 (3.73)	0.33
Race				0.14
Asian	5 (2.6%)	5 (2.9%)	0 (0.0%)	
Black	14 (7.3%)	10 (5.8%)	4 (19.0%)	
Multi-racial	27 (14.1%)	25 (14.6%)	2 (9.5%)	
Other	39 (20.3%)	37 (21.6%)	2 (9.5%)	
White	107 (55.7%)	94 (55.0%)	13 (61.9%)	
Hispanic	85 (44.3%)	81 (47.4%)	4 (19.0%)	0.014
Lives Alone	10 (5.2%)	10 (5.8%)	0 (0.0%)	0.26
Homeless	2 (1.1%)	1 (0.6%)	1 (5.0%)	0.074
Limited Activities of Daily Living	103 (53.6%)	93 (54.4%)	10 (47.6%)	0.56
Coronary Artery Disease	11 (5.7%)	10 (5.8%)	1 (4.8%)	0.84
Hypertension	57 (29.7%)	51 (29.8%)	6 (28.6%)	0.91
Cardiovascular Disease	2 (1.0%)	1 (0.6%)	1 (4.8%)	0.075
Gastroesophageal Reflux Disease	65 (33.9%)	57 (33.3%)	8 (38.1%)	0.66
Cerebral Vascular Accident	3 (1.6%)	1 (0.6%)	2 (9.5%)	0.002
Myocardial Infarction	2 (1.0%)	2 (1.2%)	0 (0.0%)	0.62
Depression/Anxiety	63 (32.8%)	54 (31.6%)	9 (42.9%)	0.3
Indication				0.55
Bronchiectasis	5 (2.6%)	5 (2.9%)	0 (0.0%)	
Cystic Fibrosis	21 (10.9%)	19 (11.1%)	2 (9.5%)	
COPD	40 (20.8%)	34 (19.9%)	6 (28.6%)	
Interstitial Lung Disease	111 (57.8%)	101 (59.1%)	10 (47.6%)	
Pulmonary Hypertension	15 (7.8%)	12 (7.0%)	3 (14.3%)	
Type of Transplant				0.61
Double	109 (56.8%)	96 (56.1%)	13 (61.9%)	
Single	83 (43.2%)	75 (43.9%)	8 (38.1%)	
SIPAT Score, median (IQR)	9 (6, 15)	9 (5.5, 15)	11 (7, 16)	0.31
Lung Allocation Score, median (IQR)	47 (37, 66)	47 (37, 66)	42 (35, 64)	0.53
Wait time, days	190 (55, 449)	195 (55, 471)	124 (41, 201)	0.27

IQR, interquartile range; SD, standard deviation.

**Table 3:** Post-Transplant Hospital Course

Factor	Total *N* = 192	No Social Readmission *N* = 171	Social Readmission *N* = 21	*p*-value
Index Hospital Length of Stay, median (IQR)	16 (13, 26.5)	16 (13, 28)	16 (14, 21)	0.88
Hospitalized before Transplant	40 (20.8%)	38 (22.2%)	2 (9.5%)	0.18
Extracorporeal Membrane Oxygenation	54 (28.1%)	48 (28.1%)	6 (28.6%)	0.96
Reoperation	12 (6.2%)	11 (6.4%)	1 (4.8%)	0.77
Reintubation	17 (8.9%)	15 (8.8%)	2 (9.5%)	0.91
New Diabetes	84 (43.8%)	74 (43.3%)	10 (47.6%)	0.7
Pneumonia	38 (19.8%)	31 (18.1%)	7 (33.3%)	0.099
Acute Respiratory Distress Syndrome	1 (0.5%)	1 (0.6%)	0 (0.0%)	0.73
Renal Insufficiency	59 (30.7%)	54 (31.6%)	5 (23.8%)	0.47
Urinary Tract Infection	4 (2.1%)	4 (2.3%)	0 (0.0%)	0.48
Deep Vein Thrombosis	2 (1.0%)	2 (1.2%)	0 (0.0%)	0.62
Ileus	16 (8.3%)	13 (7.6%)	3 (14.3%)	0.3
Gastroparesis	3 (1.6%)	3 (1.8%)	0 (0.0%)	0.54
New Atrial Arrythmia	48 (25.0%)	42 (24.6%)	6 (28.6%)	0.69
Ventricular Arrythmia	3 (1.6%)	2 (1.2%)	1 (4.8%)	0.21
Cardiogenic Shock	17 (8.9%)	15 (8.8%)	2 (9.5%)	0.91
Stroke	7 (3.6%)	7 (4.1%)	0 (0.0%)	0.34
Delayed Graft Function	31 (16.1%)	29 (17.0%)	2 (9.5%)	0.38
Pneumonia	38 (19.8%)	31 (18.1%)	7 (33.3%)	0.099
Renal Insufficiency	59 (30.7%)	54 (31.6%)	5 (23.8%)	0.47
Post-op Length of Stay, median (IQR)	16 (13, 24)	16 (13, 24)	16 (14, 21)	0.8
Discharged to home	139 (72.4%)	124 (72.5%)	15 (71.4%)	0.92

IQR, interquartile range.

Among the 192 included transplant patients, 169 (88%) were evaluated by a social worker and assigned an SIPAT score: 49 (29%) scored between 0 and 6, 105 (62%) scored between 7 and 20, and 15 (9%) scored 21 or greater. The median LAS for all included patients was 47.

### Social readmissions

Inter-rater agreement for categorizing a readmission was 95% with an associated *k* of 0.91. A total of 436 readmissions occurred within 1-year of transplant among the 192 included patients. There were a total of 33 (7.6%) social readmissions. These readmissions were classified based on care team documentation into seven categories: maladjustment (9, 27%), appointment/care compliance (7, 21%), insurance (6, 18%), medication compliance (4, 12%), support personnel (4, 12%), housing instability (2, 6%), and financial insecurity (1, 3%) (Supplementary Figure 1**;** Supplementary Table 1). Patients with maladjustment were admitted due to overwhelming anxiety related to transplant, bereavement, and substance use disorders.

At the patient level, a social readmission occurred in 21 (10.6%) of the 192 post-transplant patients. 171 patients did not experience a social readmission. There were no significant differences in pre-operative characteristics or post-transplant hospital course between patients with and without social readmissions except for Hispanic ethnicity, which was less common among patients with a social readmission (19% vs 47%; *p* = 0.01).

**Figure 2:**
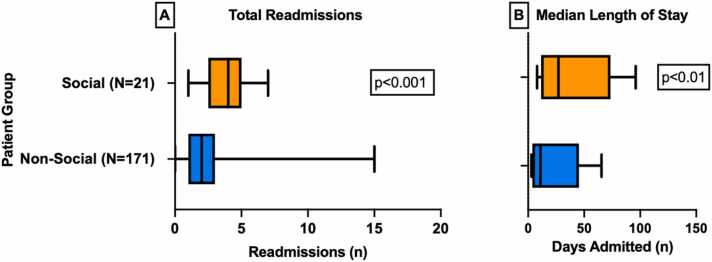
Comparisons of clinical course differences between social and non-social patient groups **(A)** total number of readmissions and **(B)** median length of stay per readmission.

In the 21 patients with a social readmission, 9 (43%) had multiple social readmissions. The median number of readmissions for patients with no social readmissions was 2 (IQR 1, 3) versus a median of 4 (IQR 2, 5) for the group with a social readmission (*p* < 0.001). The median length of stay per readmission for patients with no social readmissions was 11 days (IQR 5, 25) versus a median of 27 days (IQR 15, 51) (*p* < 0.001) (Figure 2). The median SIPAT score for the group with no social readmissions was 9 (range 6 to 15) versus a median of 11 (range 7 to 16) for the group with a social readmission (*p* = 0.31). The median LAS for the group with no social readmissions was 47 (range 37 to 66) versus a median of 42 (range 35 to 64) for the group with a social readmission (*p* = 0.53).

**Figure 3:**
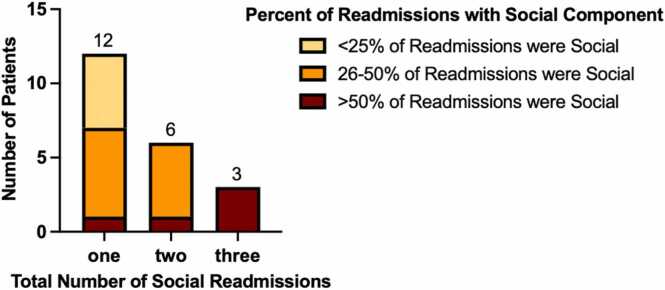
Breakdown of social vs non-social readmissions for patients with 1 vs 2 vs 3 social readmissions.

For the 21 patients with a social readmission, 12 (57%) had one social readmission, six (29%) had two social readmissions, and three (14%) had three social readmissions. For the group with one social readmission, social readmissions made up <25% of readmissions for five patients, 26%-50% of readmissions for six patients, and >50% of readmissions for one patient. For the group with two social readmissions, social readmissions made up 26%-50% of readmissions for five patients and >50% of readmissions for one patient. For the group with three social readmissions, social readmissions made up >50% of readmissions for all three patients (Figure 3). The percentage of admissions that are social (versus non-social) make up a higher percentage of a patient’s total readmissions the more social readmissions the patient has.

A social readmission represented the first or second post-operative readmission in 17 (81%) of patients with a social readmission. The timing of all social admissions in relation to non-social readmission is included in Supplementary Table 2. In time-to-event analysis, median time to readmission for patients with a social readmission was 46 days compared to 81 days in the overall population (*p* = 0.02) (Supplementary Figure 2). When excluding patients who had at least one non-social readmission from the comparator group, this trend lost its significance (46 days vs 68.5 days; *p* = 0.71) (Supplementary Figure 3).

### Burden of social readmissions

Cost (net revenue, variable cost, and contribution margin) was analyzed for 436 readmissions. Thirty-two additional readmissions were excluded from analysis due to incomplete cost data.

At the readmission level (comparing a non-social readmission to a social readmission), the median number of days spent in the hospital for non-social readmissions was 5 days versus 8 days for social readmissions (*p* = 0.004). The median net revenue for non-social readmissions was $16,921 (IQR $8649, $39,698) versus $18,862 (IQR $11,223, $47,992) for social readmissions (*p* = 0.52). The median contribution margin was $2287 (IQR −$5669, $14,661) for non-social readmissions versus −$6145 (IQR -$20,933, −$746) for social readmissions (*p* < 0.001). The median variable cost for non-social readmissions was $14,245 (IQR $8185, $31,980) versus $23,814 (IQR $11,367, $71,595) for social readmissions (*p* = 0.04).

**Figure 4:**
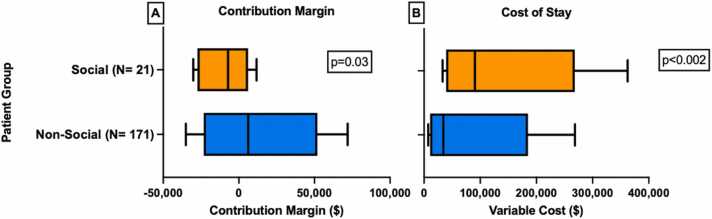
Comparison of costs incurred between social and non-social patient groups **(A)** contribution margin (the amount of money the hospital gained or lost from a given readmission) and **(B)** variable cost (costs include nursing care, room and board, and supplies, but does not include physician or advanced practice provider (APP) salaries) per stay. 10th, 90th, and median indicated with lines; the interquartile range (25th to the 75th) is represented by the box length.

At the patient level, the total median net revenue for the non-social group was $38,380 (IQR $17,340, $99,726) versus a median of $65,959 (IQR $41,312, $145,300) for the social group (*p* = 0.01). The median total variable cost for the non-social group was $34,419 (IQR $14,203, $101,429) versus $90,596 (IQR $45,487, $174,091) for the social group (*p* < 0.002). The total median contribution margin for the non-social group was $6203 (IQR −$11,331, $31,922) versus −$7161 (IQR −$24,637, $589) for the social group (*p* = 0.03) (Figure 4, Table 4).

**Table 4:** Cost Data At the Patient Level

Factor	Patients With No Social Readmissions	Patients With Social Readmissions	*p*-value
	*N* = 140	*N* = 21	
Days, median (IQR)	11 (4, 25)	27 (15, 51.12509)	0.001
Net Revenue, median (IQR)	38,380 (17,340, 99,726)	65,959 (41,312, 145,300)	0.013
Variable Cost, median (IQR)	34,419 (14,203, 101,429)	90,596 (45,487, 174,091)	0.002
Contribution Margin, median (IQR)	6203 (−11,331, 31,922)	−7161 (−24,637, 589)	0.028

IQR, interquartile range.

Insurance type was accounted for in cost analysis and did not have a statistically significant difference between the social and non-social groups. Medicaid was billed for 141 (35%) non-social readmissions and 12 (36.4%) social readmissions. Medicare was billed for 199 (49.4%) non-social readmissions and 18 (54.5%) social readmissions. Private insurance made up 61 (15.1%) non-social readmissions and 3 (9.1%) social readmissions.

## Discussion

In this study of 192 transplant patients, 11% of patients had at least one admission associated with social factors. Having one social readmission makes it more likely a patient will have more readmissions, and specifically more social readmissions. For most patients with at least one social readmission, their first social readmission occurred early in the post-operative period. Fiscally, at both the readmission and patient levels, social readmissions are longer, cost the hospital more, and garner a lower margin.

Previous studies report common reasons for readmission after lung transplant but focus primarily on comorbidities and complications.^9^, ^10^, ^11^, ^12^, ^13^ This study is important because it identifies social factors as significant and potentially modifiable, risk factors for readmission. Social contributions to adverse outcomes and readmissions have been studied in other patient populations, such as those receiving continuous flow left ventricular assist devices, and patients with higher psychosocial burden were found to be at increased risk for both adverse outcomes and readmissions.^14^ Previous studies have also started to investigate psychosocial causes for increased incidence of readmission after general surgery specifically.^15^, ^16^, ^17^ Studies investigating kidney transplantation have shown similar results in the association of psychosocial factors increasing the risk of future readmissions.^18^

SIPAT score, meant to be an objective assessment of psychosocial readiness for transplant, is one variable considered in determining a patient’s candidacy for transplant. Our study did not compare our cohort to patients deemed ineligible for transplant; however, patients are deemed ineligible for transplant at our center because of inadequate readiness, social support, psychological support, or substance use. It is,however, worth noting that the SIPAT pre-transplant scores did not significantly differ between patients with and without a social readmission. Previous studies have linked an elevated SIPAT with a higher number of readmissions.^11^ As a widely used tool, further studying the relationship between SIPAT score and clinical and fiscal outcomes could prove valuable as we strive to decrease the number of social readmissions in the post-lung-transplant population.

Patients are provided with intensive preoperative and postoperative education with their nurse, pulmonologist, and social worker. Despite efforts to assess for social readiness for transplant and efforts to educate patients on their own care, we still found readmissions related to social factors.

Social readmissions in general—and repeated social readmissions in particular—place a burden on patients and the financial well-being of the healthcare system. Clinically, we have shown that patients with social readmissions beget more social readmissions and more readmissions overall. Future initiatives should be explored to reduce readmissions after transplantation, as programs have been trialed with success in various cohorts.^19^, ^20^, ^21^ Currently, our institution has no standardized approach for addressing a social readmission or a targeted mechanism to prevent additional social readmissions. The authors are enacting a new study with an intervention aimed at ameliorating and proactively addressing social contributors to readmission through weekly phone interviews. The hope is that these interviews lead to early identification of impending social readmissions, which can then be relayed to the transplant team to identify an appropriate outpatient intervention. We acknowledge that there is significant heterogeneity behind social readmissions and that each underlying cause may require a unique intervention.

Limitations to this study include inherent concerns regarding generalizability. The LAS score at the time of transplant was relatively high at our center, reflecting the competitiveness of our region. Severity of illness and the amount of time spent on the transplant waitlist may affect the likelihood of future social readmissions; however, we did not see this in our regression (Table 2). While we suspect that our institution is not unique in its treatment of patients with major social barriers, some of these issues may be related to local availability of social safety net programs. Additionally, some psychosocial barriers identified in this study may be specific to our locality in the United States, where there is not established universal health care. Unfortunately, patients may face barriers in accessing treatment and necessary medications due to the lack of adequate health insurance coverage as the system is currently designed. These social factors may not be as prevalent in countries with different health care structures.

Additionally, social contributors to readmission were determined through chart review and were reliant on patient disclosure as well as accurate documentation. The documentation of social components to readmission is likely to be underperformed, and the true number of readmissions may be substantially higher. Identifying social readmissions in this study required a tedious and in-depth chart review and was dependent on the interpretation of a novel definition of social readmission; however, we do not believe this data is accurately captured in administrative coding, and this mechanism of chart review is critical. Despite high inter-rater reliability and *k,* it is possible that events were missed or categorized incorrectly. Due to the need for chart review, we are unable to blind this study, and it is possible reviewer bias was present, although we went to extensive lengths to prevent this with multiple independent reviews. Had we chosen to evaluate for a specific non-medical reason for readmission, eg, lack of transportation to critical monitoring visit, we may have everted some of the challenge inherent in a broader definition of social readmission. However, we were interested in non-medical readmissions in general, for which a specific variable did not exist. Additionally, any patients admitted to an outside hospital and not transferred to the study site were not captured by this study. It is extremely rare for the patients in this population to not receive transfer to the study site for treatment, but is a limitation of the study. We do not have information on career status, although the majority of patients were on disability prior to transplant and in the immediate post-transplant phase of care. Employment may influence the rate of social readmission, but this was not captured. We used readmissions within 1 year of transplant due to heterogeneity in length of stay, which we included as a covariate. In sensitivity analysis using date of discharge, we did not see any significant quantitative differences. Other limitations include heterogeneity in the hospital billing system, which varied throughout the study period. Total costs to the hospital are underestimated, as variable cost does not account for physician and advanced practice provider salary. The cost data is dependent on the accuracy of the hospital billing department. While there is strong financial incentive for accuracy, we have no external mechanism for checking the accuracy of the provided financial data.

## Conclusion

We found an association between social readmissions and increased hospital costs, length of hospital stays, and number of readmissions. A first-time social readmission should spur intervention and mobilization of resources to reduce repeated events. Given the high cost of social readmissions, investment in such a program is likely to reduce overall healthcare costs.

## Financial Disclosure Statement

The authors have no disclosures.

## Declaration of Competing Interest

The authors declare that they have no known competing financial interests or personal relationships that could have appeared to influence the work reported in this paper.

## Footnotes

Appendix A Supplementary data associated with this article can be found in the online version at doi:10.1016/j.jhlto.2025.100300.
