# Supplementary material for: Social contributions as risk factors for readmissions after lung transplantation: Clinical and financial implications
Source: JHLT Open. 2025 May 26;9:100300. doi: 10.1016/j.jhlto.2025.100300 (PMC12173132; doi:10.1016/j.jhlto.2025.100300)

Supplementary Figure 1. Social Readmission Categories


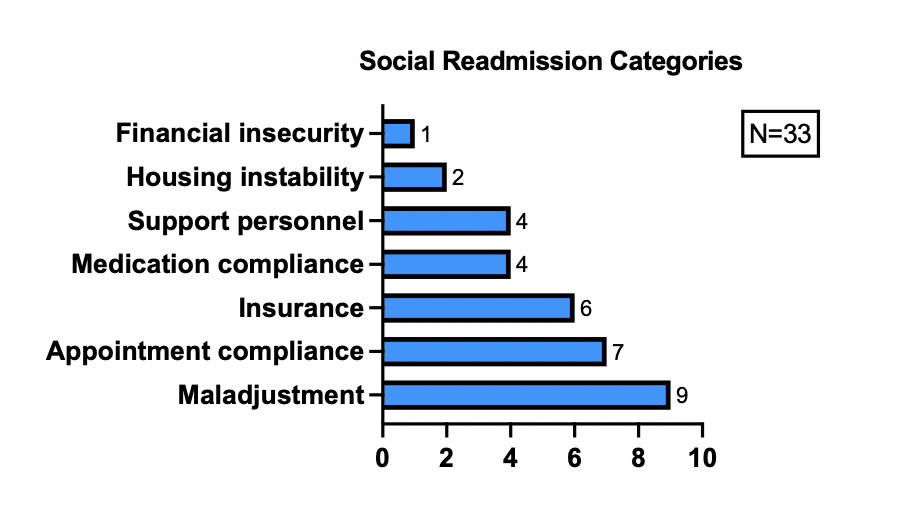


Supplementary Figure 2. Patients with social readmission(s) compared to the overall population


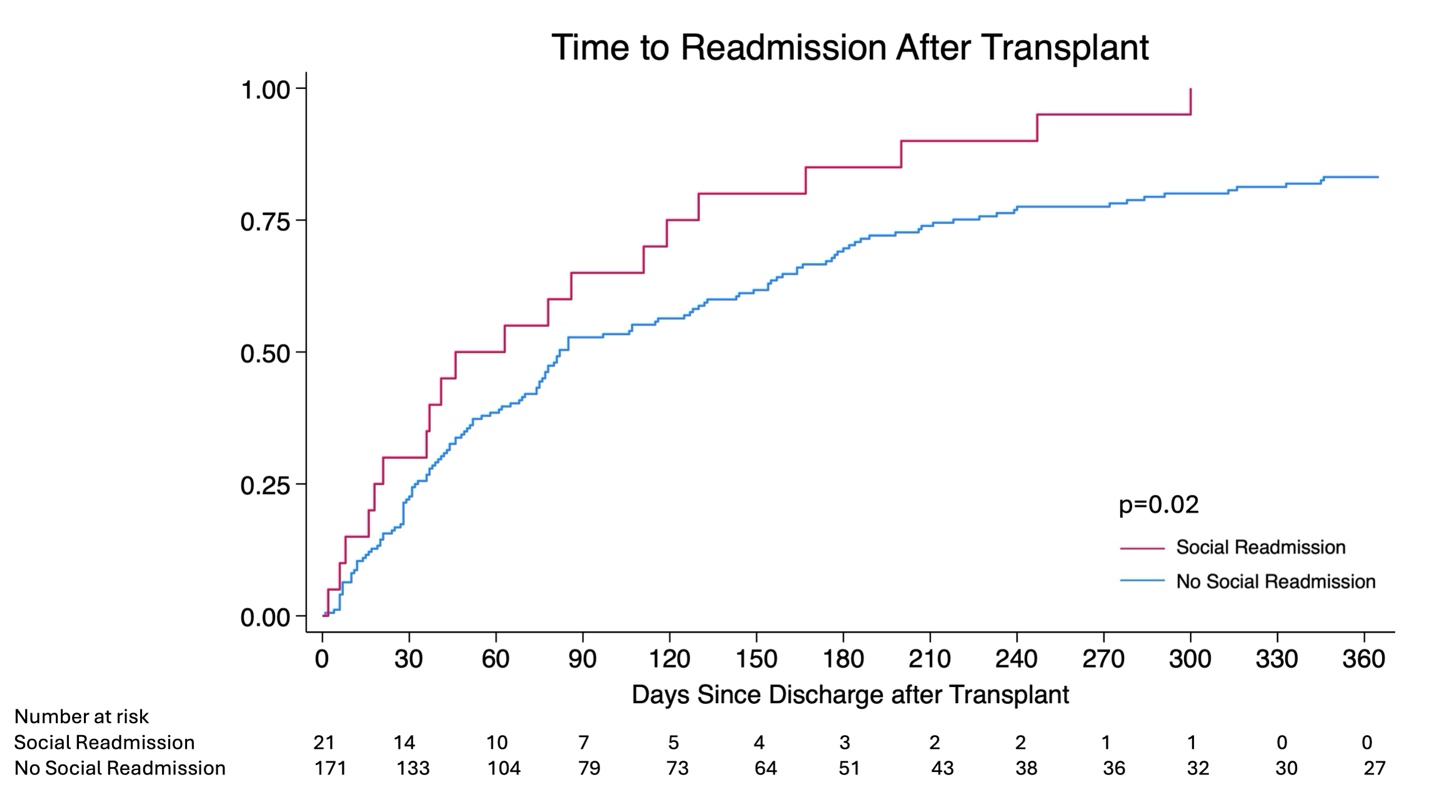


Supplementary Figure 3. Patients with social readmission(s) compared to patients with only non-social readmission(s)


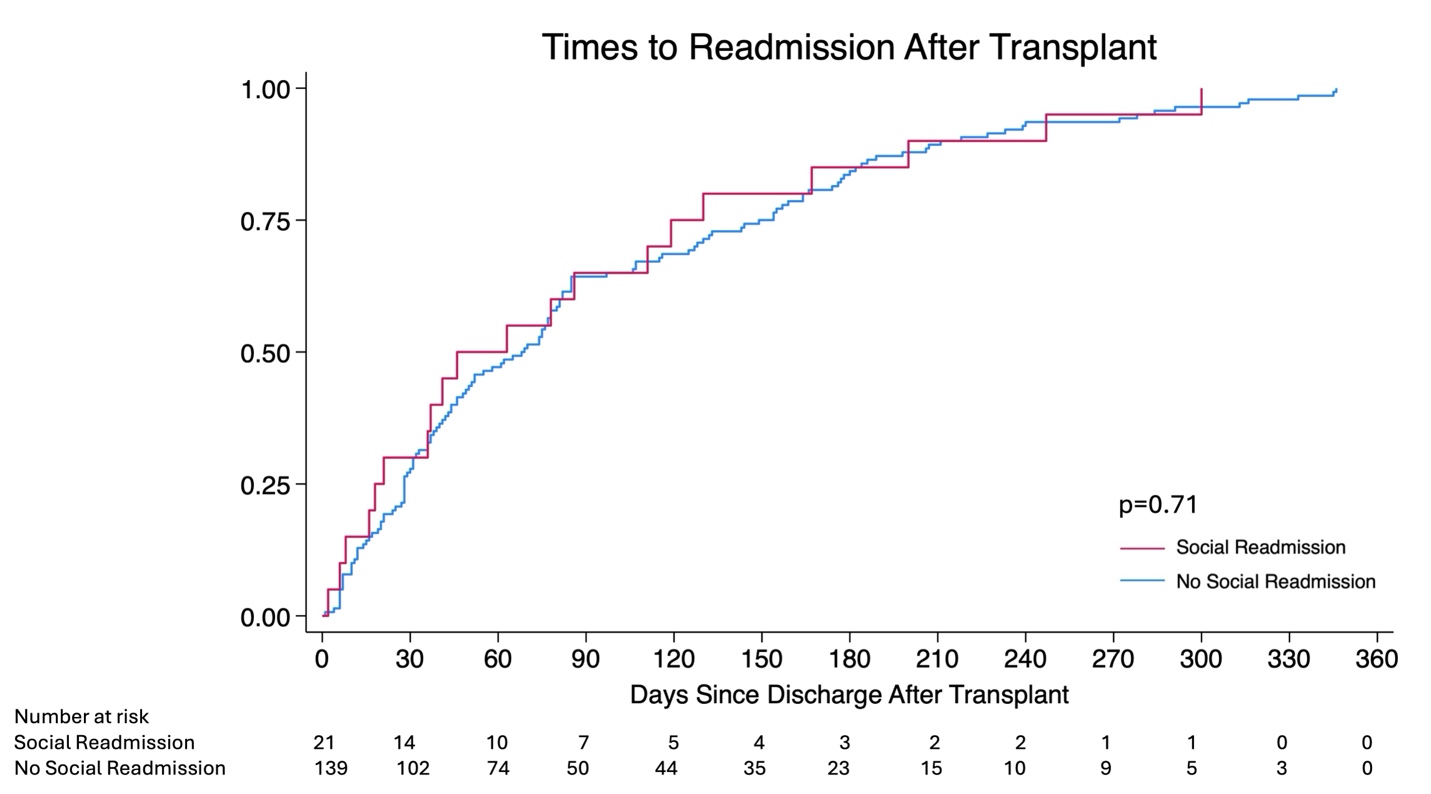

Supplement: Supplementary file 1 — Supplementary material [file mmc1.docx]
