# Supplementary material for: Social contributions as risk factors for readmissions after lung transplantation: Clinical and financial implications
Source: JHLT Open. 2025 May 26;9:100300. doi: 10.1016/j.jhlto.2025.100300 (PMC12173132; doi:10.1016/j.jhlto.2025.100300)
Supplement: Supplementary file 2 — Supplementary material [file mmc2.docx]

Supplementary Table 1: Detailed explanation of social readmission

| **Category** | **Detailed Explanation** |
| --- | --- |
| Appointment/Care Compliance | Refused home health leading to complication resulting in readmission |
| Appointment/Care Compliance | Missed follow-up appointment leading to medication mismanagement and readmission |
| Appointment/Care Compliance | Left hospital against medical advice leading to return to hospital in 24 hours |
| Appointment/Care Compliance | Non-adherent to recommended diet resulting in exacerbation of underlying comorbidities and readmission |
| Appointment/Care Compliance | Refused physical therapy leading to worsening frailty and readmission |
| Appointment/Care Compliance | Non-adherent to smoking cessation plan resulting in graft dysfunction and readmission |
| Appointment/Care Compliance | Missed follow-up appointment leading to medication errors and readmission |
| Insurance Issues | Prescribed medication not covered requiring readmission to correct |
| Insurance Issues | Denial of acute rehabilitation leading to inability patient to fail outpatient management and a readmission |
| Insurance Issues | Prescribed medication not covered requiring readmission to correct |
| Insurance Issues | Prescribed medication not covered requiring readmission to correct |
| Insurance Issues | Prescribed medication not covered requiring readmission to correct |
| Insurance Issues | At-home oxygen not covered resulting in hypoxemia and readmission |
| Medication Compliance | Stopped taking immunosuppression resulting in rejection and readmission |
| Medication Compliance | Patient taking insufficient dose of immunosuppressant despite education for increase resulting in rejection and readmission |
| Medication Compliance | Non-adherent to diabetic medication leading to hyperglycemia and readmission |
| Medication Compliance | Non-adherent to diabetic medication leading to hyperglycemia and readmission |
| Support Personnel | Primary caregiver unreliable, resulting in failure of outpatient management and readmission |
| Support Personnel | Change in social dynamics of family and caregiving leading to failure of outpatient management and readmission |
| Support Personnel | Family struggling with adequate caregiving but nonamenable to SNF, resulting in failure of outpatient management and readmission |
| Support Personnel | Insufficient care overnight leading to high-risk of fall and readmission |
| Housing Instability | Living in motel without caregiver support leading to failure of outpatient management and readmission |
| Housing Instability | Living in motel without caregiver support leading to failure of outpatient management and readmission |
| Financial Insecurity | IHSS disbursement insufficient for caregiver need leading to failure of outpatient management and readmission |
| Maladjustment | Overwhelmed by outpatient management, exacerbating underlying psychiatric disorder requiring readmission |
| Maladjustment | Steroid-induced delusions |
| Maladjustment | Relapse of substance use disorder leading to failure of outpatient management and readmission |
| Maladjustment | Non-adherent to exercise regimen due to perceived weakness leading to failure of outpatient management and readmission |
| Maladjustment | Symptomatic pain out of proportion to medical condition leading to failure of outpatient management and readmission |
| Maladjustment | Grief over loss of family members resulting in complete activities of daily living and resulting in readmission |
| Maladjustment | Overwhelmed by outpatient management, exacerbating underlying psychiatric disorder requiring readmission |
| Maladjustment | Increased alcohol consumption leading to failure of outpatient management and readmission |
| Maladjustment | Increased alcohol consumption leading to failure of outpatient management and readmission |
