# Supplementary material for: Social contributions as risk factors for readmissions after lung transplantation: Clinical and financial implications
Source: JHLT Open. 2025 May 26;9:100300. doi: 10.1016/j.jhlto.2025.100300 (PMC12173132; doi:10.1016/j.jhlto.2025.100300)
Supplement: Supplementary file 3 — Supplementary material [file mmc3.docx]

| Supplementary Table 2: Order and Type of Readmission Among 21 Patients with a Social Admissions | | | | | | | |
| --- | --- | --- | --- | --- | --- | --- | --- |
| Patient | Readmission #1 | Readmission #2 | Readmission #3 | Readmission #4 | Readmission #5 | Readmission #6 | Readmission #7 |
| 1 | Non-Social | Social | Social | Non-Social | NA | NA | NA |
| 2 | Non-Social | Social | Social | Social | NA | NA | NA |
| 3 | Non-Social | Social | Non-Social | NA | NA | NA | NA |
| 4 | Social | Non-Social | Social | Non-Social | Non-Social | NA | NA |
| 5 | Non-Social | Non-Social | Social | Non-Social | Non-Social | NA | NA |
| 6 | Non-Social | Social | NA | NA | NA | NA | NA |
| 7 | Non-Social | Social | Social | Social | NA | NA | NA |
| 8 | Non-Social | Social | Non-Social | Social | Non-Social | NA | NA |
| 9 | Non-Social | Non-Social | Social | Non-Social | Non-Social | Social | NA |
| 10 | Social | Non-Social | NA | NA | NA | NA | NA |
| 11 | Social | Non-Social | Non-Social | Non-Social | Non-Social | Non-Social | Non-Social |
| 12 | Social | Social | Social | NA | NA | NA | NA |
| 13 | Non-Social | Social | Non-Social | NA | NA | NA | NA |
| 14 | Social | Social | NA | NA | NA | NA | NA |
| 15 | Non-Social | Social | Social | Non-Social | NA | NA | NA |
| 16 | Non-Social | Non-Social | Non-Social | Social | Non-Social | Non-Social | NA |
| 17 | Non-Social | Social | NA | NA | NA | NA | NA |
| 18 | Social | NA | NA | NA | NA | NA | NA |
| 19 | Non-Social | Social | Non-Social | Non-Social | Non-Social | Non-Social | NA |
| 20 | Non-Social | Non-Social | Social | NA | NA | NA | NA |
| 21 | Social | Non-Social | NA | NA | NA | NA | NA |
| NA, not applicable (patient did not have further readmissions) | | | | | | | |
